# Enhanced Blood Clotting After Rewarming From Experimental Hypothermia in an Intact Porcine Model

**DOI:** 10.3389/fphys.2022.901908

**Published:** 2022-04-29

**Authors:** Torstein Schanche, Ole Magnus Filseth, Bjarne Østerud, Timofei V. Kondratiev, Gary C. Sieck, Torkjel Tveita

**Affiliations:** ^1^ Department of Clinical Medicine, Anaesthesia and Critical Care Research Group, UiT The Arctic University of Norway, Tromsø, Norway; ^2^ Department of Physiology and Biomedical Engineering, Mayo Clinic, Rochester, MN, United States; ^3^ Division of Surgical Medicine and Intensive Care, University Hospital of North Norway, Tromsø, Norway; ^4^ Thrombosis Research Center, Faculty of Health Sciences, UiT The Arctic University of Norway, Tromsø, Norway

**Keywords:** accidental hypothermia, rewarming, blood clotting time, blood platelets, bleeding

## Abstract

**Introduction:** Due to functional alterations of blood platelets and coagulation enzymes at low temperatures, excessive bleeding is a well-recognized complication in victims of accidental hypothermia and may present a great clinical challenge. Still, it remains largely unknown if hemostatic function normalizes upon rewarming. The aim of this study was to investigate effects of hypothermia and rewarming on blood coagulation in an intact porcine model.

**Methods:** The animals were randomized to cooling and rewarming (*n* = 10), or to serve as normothermic, time-matched controls (*n* = 3). Animals in the hypothermic group were immersion cooled in ice water to 25°C, maintained at 25°C for 1 h, and rewarmed to 38°C (normal temperature in pigs) using warm water. Clotting time was assessed indirectly at different temperatures during cooling and rewarming using a whole blood coagulometer, which measures clotting time at 38°C.

**Results:** Cooling to 25°C led to a significant increase in hemoglobin, hematocrit and red blood cell count, which persisted throughout rewarming. Cooling also caused a transiently decreased white blood cell count that returned to baseline levels upon rewarming. After rewarming from hypothermia, clotting time was significantly shortened compared to pre-hypothermic baseline values. In addition, platelet count was significantly increased.

**Discussion/Conclusion:** We found that clotting time was significantly reduced after rewarming from hypothermia. This may indicate that rewarming from severe hypothermia induces a hypercoagulable state, in which thrombus formation is more likely to occur.

## Introduction

Hypothermia is associated with impaired hemostatic function and increased mortality in the bleeding patient (Brown et al., 2012). In trauma victims, the presence of accidental hypothermia significantly increases risk of mortality (Tsuei and Kearney, 2004). At core temperatures below 32°C**,** mortality of trauma-related hypothermia is reported to be 50–100%, compared to 21–26% in non-bleeding patients with primary accidental hypothermia (Jurkovich et al., 1987; Matsuyama et al., 2018; Tsuei and Kearney, 2004; Wallner et al., 2020)**.** In the perioperative setting, hypothermia significantly increases surgical blood loss and the relative risk of transfusion requirement (Rajagopalan et al., 2008).

*In vitro* studies of the effects of hypothermia on coagulation report a mild bleeding diathesis caused by platelet adhesion defects during cooling to 33°C, (Wolberg et al., 2004), while further cooling progressively impairs hemostasis due to both reduced platelet function and clotting factor enzyme activity at low temperatures (Polderman, 2012; Wallner et al., 2020; Watts et al., 1998; Wolberg et al., 2004). However, other investigators report a hypothermia-induced increase in platelet aggregation and activation, suggesting that cooling might lead to a hypercoagulable state (Horioka et al., 2019; Xavier et al., 2007).

To detect clinically significant clotting abnormalities during hypothermia, the use of viscoelastic hemostatic assays, such as thrombelastography, have been found superior to other clotting tests like prothrombin time, activated partial thromboplastin time and activated clotting time (Martini et al., 2008). Studies using thromboelastography, and also other viscoelastic hemostatic assays, including rotational thrombelastometry and free oscillation rheometry, all report a progressive increase in clotting time at reduced body temperatures (Rundgren and Engström, 2008; Ruzicka et al., 2012; Wallner et al., 2020; Winstedt et al., 2013). These whole blood hemostatic tests measure the dynamics of clot formation and seem to reflect *in vivo* blood clotting more accurately (Horioka et al., 2020).

However, few studies have explored the effects of hypothermia and rewarming on blood clotting properties, which still remain unclear. Some investigators report the occurrence of a hypothermia-induced coagulopathy, which is completely reversible upon rewarming (Michelson et al., 1994; Valeri et al., 1987). Another study reported enhanced clotting capabilities after rewarming, resulting in thrombus formation in microvessels (Horioka et al., 2020). The aim of the present study was to investigate the impact of hypothermia and subsequent rewarming on whole blood coagulation using an intact pig model of immersion cooling and rewarming. Our model was instrumented for simultaneous measurements of global hemodynamic function, as hypothermia may lead to circulatory changes affecting blood coagulation, such as reduced cardiac output (CO) and organ blood flow (Valkov et al., 2019).

## Materials and Methods

### Animals

Thirteen castrated male juvenile pigs (20–30 kg) from a native Norwegian stock (norsk landsvin) were used in a study that was approved by the Animal Research Division at the Norwegian Food Safety Authority; reference #23/00. The approval includes strict ethical evaluation in accordance with the European Directive on the Protection of Animals used for Scientific Purposes (Directive 2010/63/EU) and the European Convention for the Protection of Vertebrate Animals used for Experimental and Other Scientific Purposes (ETS no 123, European Council). The animals received human care in accordance with The Norwegian Animal Welfare act, were penned for 3–5 days upon arrival in the animal facility, fed twice daily and had free access to water at all times. The study was carried out in compliance with the ARRIVE guidelines.

### Anesthesia and Respiratory Support

After induction of anesthesia by an intramuscular injection of ketamine hydrochloride 20 mg/kg, midazolam 25 mg and atropine 1 mg, a catheter was inserted into an ear vein and a bolus dose of fentanyl 20 µg/kg and pentobarbital sodium 10 mg/kg was given. Following tracheostomy, a continuous left femoral vein infusion of fentanyl 20 µg/kg/h, pentobarbital-sodium 4 mg/kg/h and midazolam 0.3 mg/kg/h along with Ringer’s acetate 9 ml/kg/h was started and maintained throughout the experiment. Animals were ventilated using intermittent positive pressure ventilation with an applied positive end expiratory pressure of 4 cm H_2_O (Siemens Servo 900D, Solna, Sweden). FiO_2_ was adjusted to maintain PaO_2_ > 10 kPa, and alveolar ventilation adjusted to a PaCO_2_ of 4.5–6.0 according to arterial blood gases (Chiron Diagnostics, Medfield, MA, United States), uncorrected for temperature (α-stat management).

### Hemodynamic Measurements

The left femoral artery was exposed, and a 7 F fluid filled catheter inserted for monitoring of arterial pressure and sampling of arterial blood for blood gas analysis and coagulation measurements. A 5 F Swan-Ganz thermodilution catheter (Edwards Lifesciences, Irvine, California, United States) was introduced into the right internal jugular vein and advanced to the pulmonary artery for pressure monitoring, continuous temperature recording and CO measurements. A 6 F fluid-filled pigtail catheter (Cordis Corporation, Miami, FL, United States) was inserted into the right common carotid artery and advanced to the left ventricle (LV) for LV pressure monitoring. At pre-determined temperatures, CO was measured in triplets by injecting 10 ml of precooled saline in the thermodilution catheter. Stroke volume (SV) was calculated as CO/heart rate (HR), and systemic vascular resistance (SVR) as difference between mean arterial pressure (MAP) and central venous pressure (CVP) × 80/CO. CO and SVR was indexed to body surface area (BSA), which was calculated according to the formula: BSA (m^2^) = (734 × body weight^0.656^)/10000 (Kelley et al., 1973). HR was derived from electrocardiogram monitoring using limb leads.

### Immersion Cooling and Rewarming

After instrumentation, all surgical wounds were sutured in two layers, and a tarpaulin tub surrounding the animal was filled with cold water and ice slush until 2/3 of the animal was submerged. The head was placed on a cushion and not immersed at any time during experiments. At a core temperature of 26°C, measured in the pulmonary artery, the tub was drained of water and ice slush. Subsequently, core temperature dropped to ∼ 25°C and remained stable for 1 h when exposed to room temperature. After 1 h of stable hypothermia, the animal was rewarmed by circulating hot water (38–39°C) in the tub until a core temperature of 38°C was achieved.

### Hematologic Parameters

At pre-determined temperatures, clotting time was assessed using a ReoRox^®^ 4, whole blood coagulometer (Medirox AB, Nyköping, Sweden), which measures the clotting time and elastic properties of whole blood (Tynngård, 2008; Tynngård et al., 2015). At pre-determined core temperatures, blood samples were drawn and clotting time was immediately measured in quadruplicates by equally dividing the sample into four aliquots, which were added to a four well plate. The samples were then inserted into the device without adding anticoagulants, and were set into free oscillation around its longitudinal axis with a frequency (Fq) of 10 Hz. Alterations in damping (D) and Fq of the oscillation was measured as a function of time. When blood starts to coagulate, D increases and Fq is reduced because a greater part of blood participates in the oscillation (Tynngård, 2008). Clotting time was defined as the time required for the sum of change in Fq and D to reach a preset threshold value, and was averaged for four samples. At the same temperatures, hemoglobin (Hb), white blood cell (WBC), platelet and red blood cell (RBC) counts was performed using a Cellanalyzer CA460 (Medonic AB, Stockholm, Sweden). Hct was measured in duplicates after 3 min centrifugation of Hct vials in a microcentrifuge (StatSpin III, StatSpin Technologies, MA, United States).

### Experimental Protocol

After surgical instrumentation, animals were allowed to rest for 45 min before baseline measurements were obtained. In the hypothermic group (*n* = 9), animals were cooled to 25°C, kept at 25°C for 1 h, before rewarming to 38°C. A group of normothermic, time-matched controls (*n* = 3) received the same surgical instrumentation, but were maintained at 38°C for 6 h by the use of an electrical heating mattress placed on the operating table.

### Statistical Analysis

All data are presented as the mean ± SD**.** Data distribution was tested for normality using the Shapiro-Wilk test. Changes from pre-hypothermic baseline (38°C) were assessed by one-way repeated measures analysis of variance. If the null-hypothesis was rejected, Dunnett’s method was used to compare values to baseline. If the data were not normally distributed, repeated measures analysis of variance on ranks was used. To compare differences before and after rewarming, measurements taken at 1 h of 25°C were testet against data at 38°C after rewarming, using a paired two-tailed *t*-test was used for normally distributed data and the Wilcoxon signed rank test for not normally distributed data. Changes between groups at baseline and after rewarming (38°C) were assessed using an unpaired two-tailed t-test, or alternatively the Mann-Whitney rank sum test if the data were not normally distributed. Data were analyzed and presented using SigmaPlot statistical software version 13.0 (Systat Software, San Jose, CA).

## Results

All animals survived the experimental protocol. One animal experienced ventricular fibrillation during stable hypothermia, which was subject to rapid successful defibrillation. All other animals maintained a normal sinus rhythm during cooling, hypothermia, and rewarming, as well as during stable normothermia in controls. Average rate of cooling and rewarming was 5.01°C (±0.5) and 4.88°C (±0.4) per hour, respectively.

### Hemodynamics

**FIGURE 1:**
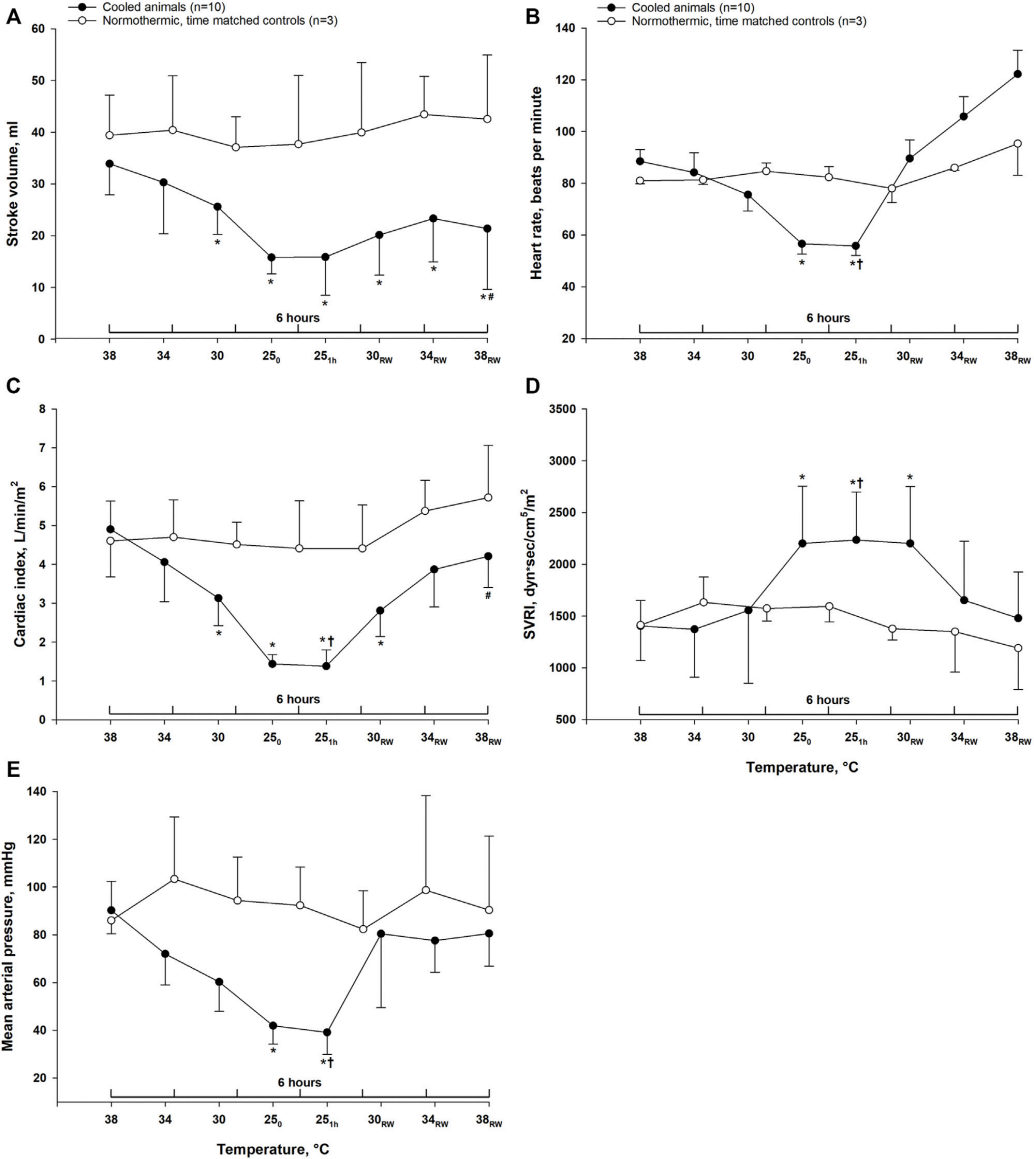
Hemodynamics. Data are presented as the mean ± SD (*n* = 10 in the hypothermia and rewarming group and *n* = 3 in the time-matched control group). **(A)** stroke volume, **(B)** heart rate, **(C)** cardiac index, **(D)** systemic vascular resistance index (SVRI), **(E)** mean arterial pressure. *p < 0.05 compared to pre-hypothermic (38°C) baseline. ^#^ *p* < 0.05 compared to time-matched normothermic control group. †*p* < 0.05 compared to post-hypothermia (38°C).

Except for the systemic vascular resistance index (SVRI), which increased significantly, cooling to 25°C led to a significant reduction of all other hemodynamic parameters (Figure 1). During the 1-h period at stable hypothermia, all variables remained unchanged. After rewarming to 38°C, MAP, SVRI, HR, and cardiac index (CI) returned to pre-hypothermic baseline values, whereas SV was significantly lowered. Compared to time-matched controls, post-hypothermic HR and CI were significantly reduced in animals after rewarming. In contrast, HR was significantly elevated after hypothermia and rewarming compared to animals in the time-matched control group. Compared to stable hypothermia (25°C 1 h), CI, HR, and MAP significantly increased after rewarming to normothermia, whereas SV remained unchanged and SVRI was significantly reduced.

### Hematologic Parameters

**FIGURE 2:**
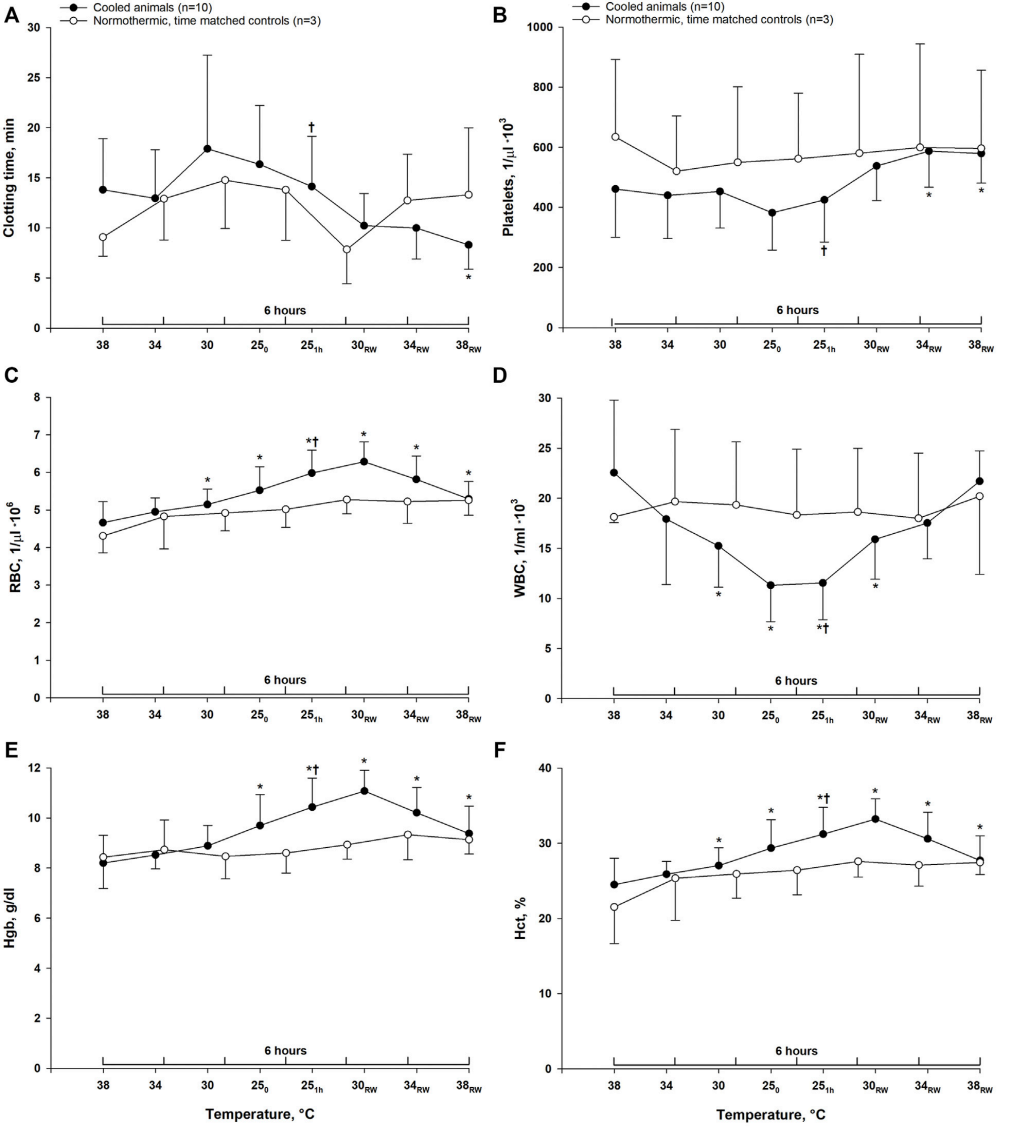
Hematologic variables. Data are presented as the mean ± SD. (*n* = 10 in the hypothermia and rewarming group and *n* = 3 in the time-matched control group) **(A)** clotting time, **(B)** platelet count, **(C)** red blood cell count, **(D)** white blood cell count, **(E)** hemoglobin (Hgb) and **(F)** hematocrit. *p < 0.05 compared to pre-hypothermic (38°C) baseline. ^†^ *p* < 0.05 compared to post-hypothermia (38°C).

Clotting time remained unchanged during cooling to 25°C and rewarming to 34°C. After rewarming to normothermia (38°C), clotting time was significantly reduced compared to both stable hypothermia (25°C) and pre-hypothermic baseline values (Figure 2). In time-matched control animals, no changes in clotting time were observed during 6-h period of stable normothermia. After cooling to 25°C, a significant reduction of WBC count was observed. Platelet count remained unchanged, whereas the number of RBC was significantly increased. After rewarming to 38°C, WBC count was significantly increased compared to stable hypothermia, and returned to pre-hypothermic baseline values, and platelet count was significantly increased compared to both stable hypothermia and pre-hypothermic baseline. Cooling led to a hemoconcentration with increased RBC count, Hct and Hb, which remained elevated throughout rewarming when compared to pre-hypothermic baseline values. Although, compared to stable hypothermia, both RBC count, Hb and Hct were significantly reduced after rewarming. In the normothermic control group, all parameters remained unchanged during 6 h of stable normothermia.

## Discussion

The main finding in this study was a significant reduction in clotting time after rewarming compared to pre-hypothermic baseline values. These findings suggest that rewarming from hypothermia might produce a hypercoagulable state. In line with our findings, Horioka et al. (2020) demonstrated enhanced whole blood coagulation along with increased levels of activated platelets after rewarming from hypothermia in an *in vivo* murine model (Horioka et al., 2020). In addition, it has been observed that refrigerated platelets become more hemostatically active upon transfusion, compared to platelets stored at room temperature (Pidcoke et al., 2014).

In the present study, clotting time was measured at 38°C irrespective of the actual body core temperature during cooling and rewarming. As such, the measured clotting time gives an indirect picture of what clotting time would be at 38°C given the current coagulation status. Measuring clotting time at true temperatures was tested in pilot experiments, but was problematic as no tendency of clotting was observed even when subjecting blood to oscillation for a prolonged period. Hence, warming of the hypothermic blood before testing will underestimate the coagulopathy of hypothermia (Forman et al., 2015). Nevertheless, our choice of methodology is in line with other studies that advocate measuring clotting time at normal body temperature regardless of true core temperature (Cundrle et al., 2013; Jeppesen et al., 2016).

Despite observations that hypothermia leads to an increased tendency of bleeding, several studies have reported increased platelet activation and aggregation at lower temperatures (Elis et al., 2002; Hall et al., 2005; Kander et al., 2014; Lindenblatt et al., 2005; Scharbert et al., 2010; Straub et al., 2006, 2011; Xavier et al., 2007). Horioka et al. found that hypothermia induced activation of platelets in the splenic pool, followed by increased levels of activated platelets in peripheral blood upon rewarming (Horioka et al., 2020, 2019). Interestingly, levels of activated platelets in peripheral blood were unchanged in asplenic mice (Horioka et al., 2020). Results from other studies suggest that hypothermia generates a minor increase in spontaneous platelet activation, whereas it significantly increases the ability of platelets to respond to activating stimuli in the form of agonists, particularly ADP (Straub et al., 2011; Van Poucke et al., 2014). These observations have led some researchers to conclude that the cause of hypothermia-induced coagulopathy is more likely a reduced availability of platelet activators, rather than intrinsic defects in platelet function at low temperatures (Van Poucke et al., 2014).

Hypothermia and rewarming appear to affect hemostasis and hemodynamics in a multifactorial manner. Taken together, many of the changes favor a hypercoagulable state. In line with previous findings (Filseth et al., 2010; Valkov et al., 2019), the current data demonstrate a markedly depressed circulatory function during hypothermia. Such low-flow conditions can promote reversible adhesion of platelets and WBCs to the vessel wall (de Vrij et al., 2021; Russell et al., 2003; Sands et al., 1979), and possibly also lead to platetet aggregation and secretion (Li et al., 2000). The rheological changes during hypothermia include a significant hemoconcentration, which is evident from the increased RBC count, Hct and Hb in response to cooling in our study. This concurs with previous studies, and is likely caused by loss of plasma volume due to increased fluid extravasation from the intravascular to the intersitital space at low temperatures (Farstad and Husby, 2014; Hammersborg et al., 2008). Low shear rates may lead to intravascular aggregation of RBCs, which is demonstrated in the hypothermic circulation (Lofstrom, 1959). RBC aggregation causes an exponentially increased blood viscosity (Lipowsky, 2005), aggravated by a simultaneous hemoconcentration. These conditions may further promote platelet margination towards the vessel wall and enhance shear-induced platelet aggregation (Zhang et al., 2004). A significant hemoconcentration was still present after rewarming, along with higher platelet count compared to pre-hypothermia. Also, after a hypothermia-induced reduction, rewarming returned WBC count to within pre-hypothermic values. These may be contributing factors to the observed reduction in post-hypothermic clotting time. High levels of RBCs and WBCs may increase the availability of platelet activators in the blood, and both can interact with platelets leading to subsequent activation (Li et al., 2000; Gillespie and Doctor, 2021). The observation of a reversible reduction of WBCs during cooling and rewarming is in line with previous reports (Sands et al., 1979; Shenaq et al., 1986). Similarly, although not observed in this study, several investigators report of a hypothermia-induced thrombocytopenia which is reversed by rewarming (Sands et al., 1979; Shenaq et al., 1986; De Vrij et al., 2014).

Furthermore, the expression of von Willebrand factor (vWF) in endothelial cells is higher at low temperatures, due to both temperature-dependent activity of the cleaving metalloprotease ADAMTS-13, and also enhanced expression of the transcription factor EGR1 giving a subsequent increased vWF expression (Ai and Gu, 1997; Horioka et al., 2020). Mahajan et al. (1981) described the occurrence of disseminated intravascular coagulation during rewarming of hypothermic patients (Mahajan et al., 1981), whereas Egorina and coworkers demonstrated an increased expression of tissue factor (TF) after rewarming rats exposed to severe hypothermia (Egorina et al., 2006). Although TF-expression was not measured in the present experiment, an increase would likely contribute to the observed reduction in clotting time after rewarming.

After rewarming we found a restoration of most hemodynamic parameters to within pre-hypothermic baseline values. However, SV remained significantly reduced, which may indicate the presence of a myocardial contractile dysfunction induced by hypothermia and rewarming, previously reported in several studies (Filseth et al., 2010; Han et al., 2010; Nilsen et al., 2021).

Measuring clotting time gives information about early hemostasis resulting in the initial thrombin burst (Leung et al., 2016). Thus, the presence of functional platelets is of critical importance to this parameter (Tynngård, 2008). However, reduced clotting time cannot be attributed exclusively to increased platelet activation, as it is also affected by functional variations in the enzymes of the clotting cascade (Leung et al., 2016). Therefore, future studies are warranted to elucidate mechanisms underlying effects of hypothermia on hemostasis. Furthermore, the present study is based on a small number of pigs, and there may exist differences in porcine and human coagulation that precludes extrapolation of the present data to humans (Kessler et al., 2011).

In summary, we found that rewarming from a 1-h period of hypothermia at 25°C led to a significant reduction in clotting time compared to pre-hypothermic baseline values, suggesting that rewarming from hypothermia might lead to a hypercoagulable state. A simultaneous increase in platelet count was observed after rewarming. Our data support the use of assays to monitor whole blood coagulation during rewarming from accidental hypothermia. In addition, these findings support the notion that in order to prevent excessive bleeding, any major surgical procedure should be postponed to after rewarming.

## Data Availability

The raw data supporting the conclusions of this article will be made available by the authors, without undue reservation.

## References

[B1] Ai X. Gu Y. (1997). The effect of low temperature on von Willbrand factor expression of cultured human umbilical vein endothelial cells. Zhonghua Wai Ke Za Zhi 35 (10), 597–599. 10678047

[B2] Brown D. J. A. Brugger H. Boyd J. Paal P. (2012). Accidental Hypothermia. N. Engl. J. Med. 367 (20), 1930–1938. 10.1056/NEJMra1114208 23150960

[B3] Cundrle I. Sramek V. Pavlik M. Suk P. Radouskova I. Zvonicek V. (2013). Temperature Corrected Thromboelastography in Hypothermia. Is it Necessary? Eur. J. Anaesthesiology 30 (2), 85–89. 10.1097/EJA.0b013e32835c3716 23249534

[B4] de Vrij E. L. Bouma H. R. Goris M. Weerman U. de Groot A. P. Kuipers J. (2021). Reversible Thrombocytopenia during Hibernation Originates from Storage and Release of Platelets in Liver Sinusoids. J. Comp. Physiol. B 191 (3), 603–615. 10.1007/s00360-021-01351-3 33661336 PMC8043940

[B5] De Vrij E. L. Vogelaar P. C. Goris M. Houwertjes M. C. Herwig A. Dugbartey G. J. (2014). Platelet Dynamics during Natural and Pharmacologically Induced Torpor and Forced Hypothermia. PLoS ONE 9 (4), e93218–12. 10.1371/journal.pone.0093218 24722364 PMC3982955

[B6] Egorina E. M. Sovershaev M. A. Kondratiev T. V. Olsen J. O. Tveita T. Østerud B. (2006). Induction of Monocytic Tissue Factor Expression after Rewarming from Hypothermia *In Vivo* Is Counteracted by Heat Shock in C-jun-dependent Manner. Atvb 26 (10), 2401–2406. 10.1161/01.atv.0000240519.46754.9c 16902157

[B7] Farstad M. Husby P. (2014). “Fluid Management during the Treatment of Immersion Hypothermia,” in Drowning. Editor Bierens J. J. L. M., 899–906. 10.1007/978-3-642-04253-9_138

[B8] Filseth O. M. How O.-J. Kondratiev T. Gamst T. M. Tveita T. (2010). Post-hypothermic Cardiac Left Ventricular Systolic Dysfunction after Rewarming in an Intact Pig Model. Crit. Care 14 (6), R211. 10.1186/cc9334 21092272 PMC3220015

[B9] Forman K. Wong E. Gallagher M. McCarter R. Luban N. Massaro A. (2015). Effect of Temperature on Thromboelastography (TEG) and Implications for Clinical Use in Neonates Undergoing Therapeutic Hypothermia. Persistent 73 (4), 389–400. 10.1530/ERC-14-0411 PMC3992188 24522100

[B10] Gillespie A. H. Doctor A. (2021). Red Blood Cell Contribution to Hemostasis. Front. Pediatr. 9 (April), 1–9. 10.3389/fped.2021.629824 PMC8047051 33869111

[B11] Hall M. W. Goodman P. D. Alston S. M. Solen K. A. Manor Y. Lishner M. (2002). Hypothermia-induced Platelet Aggregation in Heparinized Flowing Human Blood: Identification of a High Responder Subpopulation. Am. J. Hematol. 69 (1), 45–55. 10.1002/ajh.10018 11835331

[B12] Hall M. W. Hopkins R. O. Long J. W. Mohammad S. F. Solen K. a. (2005). Hypothermia-induced Platelet Aggregation and Cognitive Decline in Coronary Artery Bypass Surgery: a Pilot Study. Perfusion 20 (3), 157–167. 10.1191/0267659105pf814oa 16038388

[B13] Hammersborg S. M. Brekke H. K. Haugen O. Farstad M. Husby P. (2008). Surface Cooling versus Core Cooling: Comparative Studies of Microvascular Fluid- and Protein-Shifts in a Porcine Model. Resuscitation 79 (2), 292–300. 10.1016/j.resuscitation.2008.06.008 18656301

[B14] Han Y.-S. Tveita T. Prakash Y. S. Sieck G. C. (2010). Mechanisms Underlying Hypothermia-Induced Cardiac Contractile Dysfunction. Am. J. Physiology-Heart Circulatory Physiol. 298 (3), H890–H897. 10.1152/ajpheart.00805.2009 PMC7938765 20023122

[B15] Horioka K. Tanaka H. Isozaki S. Konishi H. Addo L. Takauji S. (2020). Rewarming from Accidental Hypothermia Enhances Whole Blood Clotting Properties in a Murine Model. Thromb. Res. 195 (June), 114–119. 10.1016/j.thromres.2020.07.022 32683149

[B16] Horioka K. Tanaka H. Isozaki S. Okuda K. Asari M. Shiono H. (2019). Hypothermia‐induced Activation of the Splenic Platelet Pool as a Risk Factor for Thrombotic Disease in a Mouse Model. J. Thromb. Haemost. 17 (10), 1762–1771. 10.1111/jth.14555 31237986 PMC6851562

[B17] Jeppesen A. N. Kirkegaard H. Ilkjær S. Hvas A. M. (2016). Influence of Temperature on Thromboelastometry and Platelet Aggregation in Cardiac Arrest Patients Undergoing Targeted Temperature Management. Crit. Care 20 (1), 118. 10.1186/s13054-016-1302-9 27129380 PMC4851809

[B18] Jurkovich G. J. Greiser W. B. Luterman A. Curreri P. W. (1987). Hypothermia in Trauma Victims. J. Trauma Inj. Infect. Crit. Care 27, 1019–1024. 10.1097/00005373-198709000-00011 3656464

[B19] Kander T. Dankiewicz J. Friberg H. Schött U. (2014). Platelet Aggregation and Clot Formation in Comatose Survivors of Cardiac Arrest Treated with Induced Hypothermia and Dual Platelet Inhibition with Aspirin and Ticagrelor; a Prospective Observational Study. Crit. Care 18 (5), 495. 10.1186/s13054-014-0495-z 25292183 PMC4194371

[B20] Kelley K. W. Curtis S. E. Marzan G. T. Karara H. M. Anderson C. R. (1973). Body Surface Area of Female Swine. J. Anim. Sci. 36 (5), 927–930. 10.2527/jas1973.365927x 4703721

[B21] Kessler U. Grau T. Gronchi F. Berger S. Brandt S. Bracht H. (2011). Comparison of Porcine and Human Coagulation by Thrombelastometry. Thromb. Res. 128 (5), 477–482. 10.1016/j.thromres.2011.03.013 21492909

[B22] Leung L. Manucci P. M. Timauer J. S. (2016). Overview of Hemostasis.

[B23] Li N. Hu H. Lindqvist M. Wikström-Jonsson E. Goodall A. H. Hjemdahl P. (2000). Platelet-leukocyte Cross Talk in Whole Blood. Atvb 20 (12), 2702–2708. 10.1161/01.ATV.20.12.2702 11116075

[B24] Lindenblatt N. Menger M. D. Klar E. Vollmar B. (2005). Sustained Hypothermia Accelerates Microvascular Thrombus Formation in Mice. Am. J. Physiology-Heart Circulatory Physiol. 289 (6), H2680–H2687. 10.1152/ajpheart.00425.2005 16100248

[B25] Lipowsky H. H. (2005). Microvascular Rheology and Hemodynamics. Microcirculation 12 (1), 5–15. 10.1080/10739680590894966 15804970

[B26] Löfström B. (1959). Induced Hypothermia and Intravascular Aggregation. Acta Anaesthesiol.Scand., Suppl. 3, 1–19. 10.1111/j.1399-6576.1959.tb05479.x 14417952

[B27] Mahajan S. L. Myers T. J. Baldini M. G. (1981). Disseminated Intravascular Coagulation during Rewarming Following Hypothermia. JAMA 245 (24), 2517–2518. 10.1001/jama.245.24.2517 7230491

[B28] Martini W. Z. Cortez D. S. Dubick M. A. Park M. S. Holcomb J. B. (2008). Thrombelastography Is Better Than PT, aPTT, and Activated Clotting Time in Detecting Clinically Relevant Clotting Abnormalities after Hypothermia, Hemorrhagic Shock and Resuscitation in Pigs. J. Trauma 65 (3), 535–543. 10.1097/TA.0b013e31818379a6 18784565

[B29] Matsuyama T. Morita S. Ehara N. Miyamae N. Okada Y. Jo T. (2018). Characteristics and Outcomes of Accidental Hypothermia in Japan: The J-Point Registry. Emerg. Med. J. 35 (11), 207238. 10.1136/emermed-2017-207238 29886414

[B30] Michelson A. D. MacGregor H. Barnard M. R. Kestin A. S. Rohrer M. J. Valeri C. R. (1994). Reversible Inhibition of Human Platelet Activation by Hypothermia *In Vivo* and *In Vitro* . Thromb. Haemost. 71 (5), 633–640. 10.1055/s-0038-1642495 7522354

[B31] Nilsen J. H. Schanche T. Kondratiev T. V. Hevrøy O. Sieck G. C. Tveita T. (2021). Maintaining Intravenous Volume Mitigates Hypothermia‐induced Myocardial Dysfunction and Accumulation of Intracellular Ca 2+. Exp. Physiol. 106, 1196–1207. 10.1113/EP089397 33728692

[B32] Pidcoke H. F. Spinella P. C. Ramasubramanian A. K. Strandenes G. Hervig T. Ness P. M. (2014). Refrigerated Platelets for the Treatment of Acute Bleeding. Shock 41, 51–53. 10.1097/SHK.0000000000000078 24662779

[B33] Polderman K. H. (2012). Hypothermia and Coagulation. Crit. Care Med. 16 (Suppl. 2), A20. 10.1186/cc11278

[B34] Rajagopalan S. Mascha E. Na J. Sessler D. I. (2008). The Effects of Mild Perioperative Hypothermia on Blood Loss and Transfusion Requirement. Anesthesiology 108 (1), 71–77. 10.1097/01.anes.0000296719.73450.52 18156884

[B35] Rundgren M. Engström M. (2008). A Thromboelastometric Evaluation of the Effects of Hypothermia on the Coagulation System. Anesth. Analgesia 107 (5), 1465–1468. 10.1213/ane.0b013e31817ee955 18931200

[B36] Russell J. Cooper D. Tailor A. Stokes K. Y. Granger D. N. (2003). Low Venular Shear Rates Promote Leukocyte-dependent Recruitment of Adherent Platelets. Am. J. Physiology-Gastrointestinal Liver Physiol. 284 (1 47-1), G123–G129. 10.1152/ajpgi.00303.2002 12388188

[B37] Ruzicka J. Stengl M. Bolek L. Benes J. Matejovic M. Krouzecky A. (2012). Hypothermic Anticoagulation. Blood Coagul. Fibrinolysis : Int. J. Haemost. Thromb. 23 (4), 285–289. 10.1097/MBC.0b013e328351885a 22356838

[B38] Sands M. P. Mohri H. Sato S. Mannik M. Hessel E. A. Dillard D. H. (1979). Hematorheology during Deep Hypothermia. Cryobiology 16, 229–239. 10.1016/0011-2240(79)90036-1 477368

[B39] Scharbert G. Kalb M. L. Essmeister R. Kozek-Langenecker S. a. (2010). Mild and Moderate Hypothermia Increases Platelet Aggregation Induced by Various Agonists: a Whole Bloodin Vitrostudy. Platelets 21 (1), 44–48. 10.3109/09537100903420269 19954411

[B40] Shenaq S. A. Yawn D. H. Saleem A. Joswiak R. Crawford E. S. (1986). Effect of Profound Hypothermia on Leukocytes and Platelets. Ann. Clin. Lab. Sci. 16 (2), 130–133. 3963733

[B41] Straub A. Breuer M. Wendel H. P. Karlheinz P. Dietz K. Ziemer G. (2006). Critical Temperature Ranges of Hypothermia-Induced Platelet Activation: Possible Implications for Cooling Patients in Cardiac Surgery. Thromb. Haemost. 96 (6), 756–766. 10.1160/TH06 17393024

[B42] Straub A. Krajewski S. Hohmann J. D. Westein E. Jia F. Bassler N. (2011). Evidence of Platelet Activation at Medically Used Hypothermia and Mechanistic Data Indicating ADP as a Key Mediator and Therapeutic Target. Atvb 31 (7), 1607–1616. 10.1161/ATVBAHA.111.226373 21512161

[B43] Tsuei B. J. Kearney P. A. (2004). Hypothermia in the Trauma Patient. Injury 35 (1), 7–15. 10.1016/S0020-1383(03)00309-7 14728949

[B44] Tveita T. Ytrehus K. Myhre E. S. P. Hevrøy O. (1998). Left Ventricular Dysfunction Following Rewarming from Experimental Hypothermia. J. Appl. Physiol. 85 (6), 2135–2139. 10.1152/jappl.1998.85.6.2135 9843536

[B45] Tynngård N. (2008). Free Oscillation Rheometry in the Assessment of Platelet Quality

[B46] Tynngård N. Lindahl T. L. Ramström S. (2015). Assays of Different Aspects of Haemostasis - what Do They Measure? Thromb. J 13 (1), 1–10. 10.1186/s12959-015-0036-2 25688179 PMC4329663

[B47] Valeri C. R. Shukri Khuri G. C. Feingold H. Mark G. R. Altschule S. Altschule M. D. (1987). Hypothermia-induced Reversible Platelet Dysfunction. Ann. Surg. 205 (2), 175–181. 10.1097/00000658-198702000-00012 3813688 PMC1492823

[B48] Valkov S. Mohyuddin R. Nilsen J. H. Schanche T. Kondratiev T. V. Sieck G. C. (2019). Organ Blood Flow and O2 Transport during Hypothermia (27°C) and Rewarming in a Pig Model. Exp. Physiol. 104 (1), 50–60. 10.1113/EP087205 30375081

[B49] Van Poucke S. Stevens K. Marcus A. E. Lancé M. (2014). Hypothermia: Effects on Platelet Function and Hemostasis. Thromb. J 12 (1), 31. 10.1186/s12959-014-0031-z 25506269 PMC4265340

[B50] Wallner B. Schenk B. Hermann M. Paal P. Falk M. Strapazzon G. (2020). Hypothermia-Associated Coagulopathy: A Comparison of Viscoelastic Monitoring, Platelet Function, and Real Time Live Confocal Microscopy at Low Blood Temperatures, an *In Vitro* Experimental Study. Front. Physiol. 11 (July), 1–10. 10.3389/fphys.2020.00843 32765300 PMC7381250

[B51] Watts D. D. Trask A. Soeken K. Perdue P. Dols S. Kaufmann C. (1998). Hypothermic Coagulopathy in Trauma. J. Trauma Inj. Infect. Crit. Care 44 (5), 846–854. 10.1097/00005373-199805000-00017 9603087

[B52] Winstedt D. Tynngård N. Olanders K. Schött U. (2013). Free Oscillation Rheometry Monitoring of Haemodilution and Hypothermia and Correction with Fibrinogen and Factor XIII Concentrates. Scand. J. Trauma Resusc Emerg. Med. 21 (1), 1–9. 10.1186/1757-7241-21-20 23517637 PMC3621621

[B53] Wolberg A. S. Meng Z. H. Monroe D. M. Hoffman M. (2004). A Systematic Evaluation of the Effect of Temperature on Coagulation Enzyme Activity and Platelet Function. J. Trauma Inj. Infect. Crit. Care 56 (6), 1221–1228. 10.1097/01.TA.0000064328.97941.FC 15211129

[B54] Xavier R. G. White A. E. Fox S. C. Wilcox R. G. Heptinstall S. (2007). Enhanced Platelet Aggregation and Activation under Conditions of Hypothermia. Thromb. Haemost. 98 (2), 1266–1275. 10.1160/th07-03-0189 18064324

[B55] Zhang J.-n. Wood J. Bergeron A. L. McBride L. Ball C. Yu Q. (2004). Effects of Low Temperature on Shear-Induced Platelet Aggregation and Activation. J. Trauma Inj. Infect. Crit. Care 57 (2), 216–223. 10.1097/01.TA.0000093366.98819.FE 15345964

